# Nonhuman Primate Model of Oculocutaneous Albinism with *TYR* and *OCA2* Mutations

**DOI:** 10.34133/2020/1658678

**Published:** 2020-03-11

**Authors:** Kun-Chao Wu, Ji-Neng Lv, Hui Yang, Feng-Mei Yang, Rui Lin, Qiang Lin, Ren-Juan Shen, Jun-Bin Wang, Wen-Hua Duan, Min Hu, Jun Zhang, Zhan-Long He, Zi-Bing Jin

**Affiliations:** ^1^Division of Ophthalmic Genetics, The Eye Hospital, Laboratory for Stem Cell & Retinal Regeneration, Institute of Stem Cell Research, Wenzhou Medical University, Wenzhou 325027, China; ^2^National Center for International Research in Regenerative Medicine and Neurogenetics, National Clinical Research Center for Ocular Diseases, State Key Laboratory of Ophthalmology, Optometry and Visual Science, Wenzhou 325027, China; ^3^Institute of Medical Biology, Chinese Academy of Medical Sciences, And Peking Union Medical College (CAMS & PUMC), Yunnan Key Laboratory of Vaccine Research Development on Severe Infectious Disease, Kunming 650118, China; ^4^Department of Ophthalmology, The Second People's Hospital of Yunnan Province, Fourth Affiliated Hospital of Kunming Medical University, Key Laboratory of Yunnan Province for the Prevention and Treatment of Ophthalmology, Kunming 650021, China; ^5^Laboratory of Retinal Physiology & Disease, The Eye Hospital, Wenzhou Medical University, Wenzhou 325027, China

## Abstract

Human visual acuity is anatomically determined by the retinal fovea. The ontogenetic development of the fovea can be seriously hindered by oculocutaneous albinism (OCA), which is characterized by a disorder of melanin synthesis. Although people of all ethnic backgrounds can be affected, no efficient treatments for OCA have been developed thus far, due partly to the lack of effective animal models. Rhesus macaques are genetically homologous to humans and, most importantly, exhibit structures of the macula and fovea that are similar to those of humans; thus, rhesus macaques present special advantages in the modeling and study of human macular and foveal diseases. In this study, we identified rhesus macaque models with clinical characteristics consistent with those of OCA patients according to observations of ocular behavior, fundus examination, and optical coherence tomography. Genomic sequencing revealed a biallelic p.L312I mutation in *TYR* and a homozygous p.S788L mutation in *OCA2*, both of which were further confirmed to affect melanin biosynthesis via *in vitro* assays. These rhesus macaque models of OCA will be useful animal resources for studying foveal development and for preclinical trials of new therapies for OCA.

## 1. Introduction

High spatial resolution vision in humans is anatomically determined by the retinal fovea, which is characterized by a high-density distribution of cone photoreceptors in an avascular zone [1–3]. The human fovea contains approximately 25% retinal ganglion cells, and the synaptic connections between cones, bipolar cells, and ganglion cells in a 1 : 1 : 1 ratio underlie the precise transmission of visual signals [4, 5]. The ontogenetic development of the fovea initially begins in fetal week 12, followed by the centripetal and centrifugal displacement of the cones and inner retinal layers separately at birth, and the fovea finally becomes mature at approximately 4 years postnatally [1–3]. A healthy developed fovea plays a critical role in accurate visual acuity. However, visual acuity can be seriously reduced by a disorder of melanin biosynthesis known as oculocutaneous albinism (OCA). OCA is a group of autosomal recessive disorders characterized by the loss of or a reduction in pigmentation in the eyes, hair, and skin. Populations of all ethnic backgrounds can be affected by OCA, with an estimated global prevalence of 1 : 17,000 [6–9]. Very rarely, OCA may be accompanied by systematic defects such as Hermansky-Pudlak and Chediak-Higashi syndromes [10–13]. To date, six disease-causing genes of nonsyndromic OCA have been reported [14–19], among which the *TYR* and *OCA2* genes are the most commonly implicated in OCA [20, 21]. Tyrosine, encoded by the *TYR* gene, regulates the rate-limiting step that converts L-tyrosine to L-DOPA and subsequently to dopaquinone [14, 22]. On the other hand, OCA2 is a melanosome-specific transmembrane protein that plays an important role in modulating the pH of melanosomes as well as the activity of chloride-selective anions, which are critical for maintaining the normal process of pigment formation [23, 24]. OCA is always associated with severe visual system damage, including iris translucency, congenital nystagmus, strabismus, misrouting of optic nerves, color vision defects, hypopigmentation of the retinal pigment epithelium (RPE), and foveal hypoplasia, which lead to poor vision and seriously affect the quality of life of OCA patients [6, 25–27].

The mechanisms of OCA pathogenesis have been reported in rodents, dogs, and water buffaloes [28–31]. However, these OCA animal models present the major limitation of the absence of a foveal structure similar to that of the human retina. To date, no effective treatments for OCA have been developed, due at least in part to the bottleneck of the lack of an appropriate animal model with a macula and foveal centralis. Rhesus macaques are Old World primates that present several special advantages for the study of human retinopathies. First, the phototransduction mechanism and immune microenvironment of rhesus macaques are similar to those of humans [32, 33]. Second, the intraocular structure of rhesus macaques is highly similar to that of the human eye, most notably in the presence of a macula and fovea [33]. Moreover, the color vision of rhesus monkeys makes them more comparable to humans than to New World primates such as capuchin monkeys [34, 35]. To the best of our knowledge, no systematic ocular disorders clarified by defined causative mutations of OCA have been described in rhesus macaques in previous studies.

Herein, we report rhesus macaque models with spontaneous oculocutaneous albinism. The characteristic symptoms, clinical manifestations, and functional testing results of these albinotic rhesus macaques are highly parallel to those of OCA patients. Moreover, the causative mutations were revealed by whole-genome sequencing. The pathogenicity of the candidate mutations was finally validated by *in vitro* functional assays.

## 2. Results

### 2.1. Albino NHPs Display Clinical Manifestations of OCA

**Figure 1:**
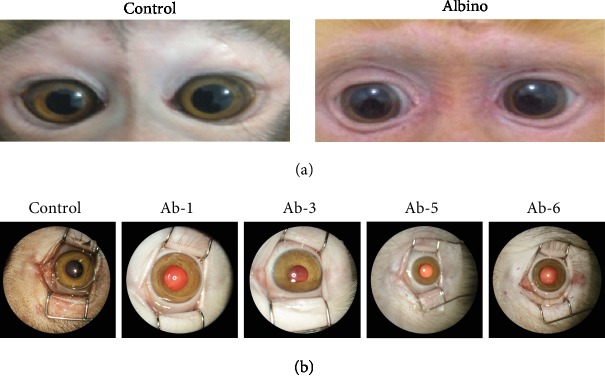
Clinical manifestations of albino rhesus macaques. (a) Representative photos of the monkeys. (b) Iris characterization and fundus reflection of albino and healthy rhesus macaques.

**Figure 2:**
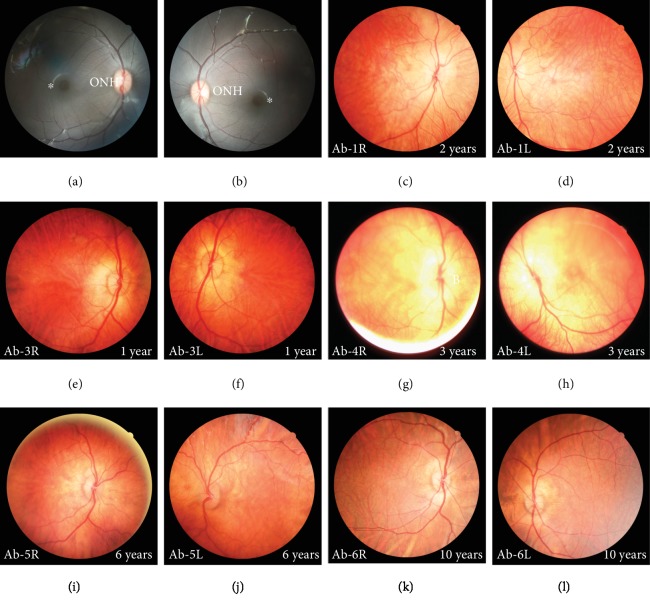
Fundography of the rhesus macaques with oculocutaneous albinism. (a, b) Representative fundi of healthy subjects. (c–l) OCA monkeys displayed extensive depigmentation. The asterisk indicates the central retina. ONH: optical nerve head.

We identified six rhesus macaques with an albino appearance characterized by red hair and pink skin (Figure 1(a)). These albino monkeys were 1-10 years old, and both male (Ab-2, Ab-3, Ab-5, and Ab-6) and female (Ab-1, Ab-4) individuals were affected. Apparent horizontal nystagmus was observed in these monkeys, which is a typical clinical behavioral characteristic of OCA patients (Table 1 and Supplementary Videos [Supplementary-material supplementary-material-1] and [Supplementary-material supplementary-material-1]). Compared with healthy individuals, the iris color was different in the albino subjects (Figure 1(b)). Anatomically, the fovea lies slightly below the center of the optic disk at the temporal fundus in healthy rhesus macaques (Figures 2(a) and 2(b)). Fundography of these albino subjects showed extensive hypopigmentation and the absence of a foveal centralis, while the choroid vasculature appeared to show the loss of pigmentation (Figures 2(c)–2(l)).

**Figure 3:**
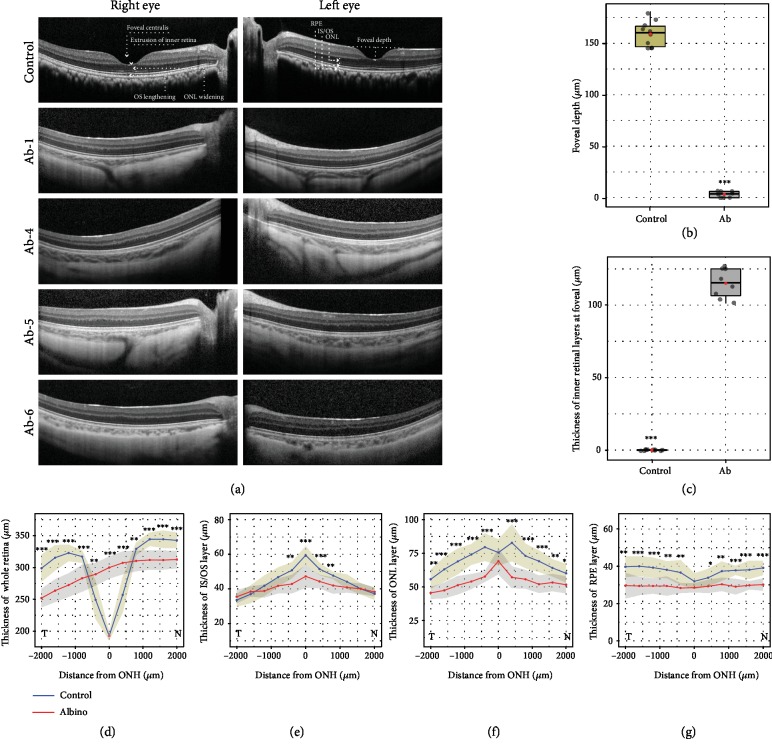
Foveal hypoplasia and retinal thickness changes in rhesus macaques with oculocutaneous albinism. (a) OCT imaging of the OCA monkeys. No obvious foveal pit was observed. ONL: outer nuclear layer; IS/OS: inner segment/outer segment; RPE: retinal epithelium cell. (b) Comparison of foveal depth between healthy individuals and albinotic subjects. (c) Statistical analysis of the thickness of the inner retinal layers between normal and albinotic rhesus macaques. The inner retinal layers include the inner nuclear layer, inner plexiform layer, retinal ganglion cell layer, and retinal nerve fiber layer. Each black plot represents the value of each eye, the red plot represents the mean value, and the black line in the boxplot represents the median value. (d, e) Thickness changes in the whole retina, ONL, IS/OS, and RPE layer in albino NHPs. The blue and red lines represent normal and albino individuals, respectively. *N* ≥ 4. ^∗^*p* < 0.05, ^∗∗^*p* < 0.01, and ^∗∗∗^*p* < 0.001.

According to the presence or absence of the extrusion of the plexiform layers, foveal pit, outer segment lengthening, and outer nuclear layer (ONL) widening, foveal hypoplasia can be divided into four grades in humans [36]. Spectral domain optical coherence tomography (SD-OCT) confirmed the absence of a foveal centralis in the albino rhesus macaques, and these albinotic subjects exhibited an obvious absence of the extrusion of the plexiform layers and outer segment lengthening (Figure 3(a)). Compared with healthy individuals, larger areas of the choroid and even sclera could be imaged, indicating extensive retinal hypopigmentation in the albino macaques (Figure 3(a)). The foveal depth in the albinotic subjects was significantly shallower than that in healthy individuals (Figure 3(b)), indicating that the characteristics of the OCA monkeys were consistent with a high degree of OCA in humans. Thicker inner retinal layers at the fovea were found in the albino subjects (Figure 3(c)). In addition, there were marked changes in the thickness of different retinal layers in the disease subjects (Figures 3(d)–3(g)), and the thinner IS/OS and ONL layers of the retina were a general indication of visual impairment. These results collectively suggest that albino rhesus macaques mimic oculocutaneous albinism in human patients.

### 2.2. Identification of Genetic Mutations in the *TYR* and *OCA2* Genes

**Figure 4:**
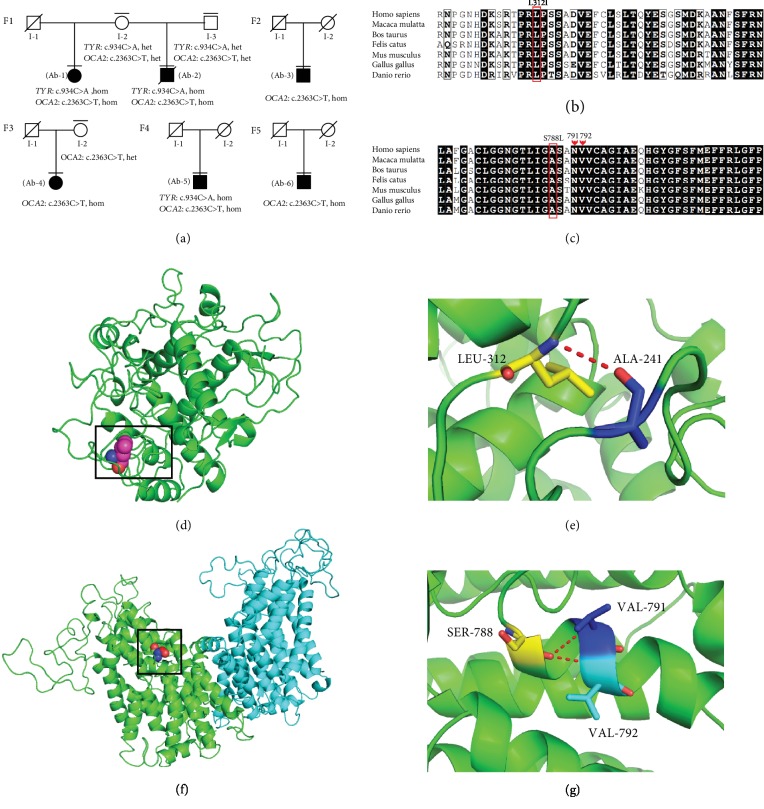
Mutation assessments. (a) Pedigree and cosegregation analysis of albino rhesus macaques. The black plot represents the OCA monkeys (Ab-1 to Ab-6). Subjects with a horizontal line underwent whole-genome sequencing. A diagonal line indicates a decrease. F: family; hom: homozygous mutation; het: heterozygous mutation. (b, c) Multiple species alignment comparison of the identified mutations (p.L312I in TYR and p.S788L in OCA2) in the albino monkeys. (d–g) 3D homology modeling of TYR and OCA2. Homology modeling showed that S788 of the OCA2 protein was likely connected to valine at positions 791 and 792, which were also highly conserved among multiple species.

Genomic DNA was extracted from six albino rhesus macaques and their available parents (Figure 4(a)), followed by whole-genome sequencing. Candidate mutations of the reported genes responsible for nonsyndromic OCA in humans were analyzed. The sequencing results showed that all of these albino monkeys carried a homozygous missense mutation, c.2363C>T, in the *OCA2* gene. Additionally, three albino subjects (Ab-1, Ab-2, and Ab-5) were found to exhibit a homozygous missense mutation, c.934C>A, in the *TYR* gene. We then performed cosegregation analysis to determine whether the mutations cosegregated with the disease in the albino monkeys and their available parents. The parents of Ab-2 were both heterozygous for the two identified mutations. In family 3, the mother of individual Ab-4 was heterozygous for the *OCA2* mutation (Figure 4(a)). To confirm whether these mutations exist in normal NHPs, we further screened the identified variants in 35 unrelated wild-type rhesus monkeys by Sanger sequencing and found that none of these monkeys carried these mutations. These results indicated that the identified mutations in the TYR and OCA2 genes partially cosegregated with OCA in an autosomal recessive manner.

The c.934C>A mutation in the *TYR* gene resulted in a change from leucine to isoleucine at position 312 of the TYR protein (p.L312I), and the c.2363C>T mutation in the *OCA2* gene led to the replacement of serine with leucine at position 788 of the OCA2 protein (p.S788L). Alignment analysis among multiple species showed that the amino acids affected by each mutation are highly conserved (Figures 4(b) and 4(c)). Notably, the *TYR* mutation found in the monkeys has been identified in OCA patients (Albinism Database, http://www.ifpcs.org/albinism/). The three-dimensional structures of the TYR and OCA2 proteins were simulated by using SwissProt and visualized by using PyMOL. The modeling results (Figures 4(d) and 4(e)) indicated that L312 of the TYR protein is likely connected to alanine at position 241, which is also highly conserved among species. The modeled structure of the OCA2 protein indicated that S788 is located at an *α*-helix and is likely connected to two other highly conserved amino acids: valine residues at positions 791 and 792 (Figures 4(f) and 4(g)). These results suggested that L312 of TYR and S788 of OCA2 might be important for these proteins. Furthermore, the computational prediction of pathogenicity showed that each identified mutation in the *TYR* or *OCA2* gene is highly pathological (Supplementary [Supplementary-material supplementary-material-1]). Considering these results together, we concluded that the identified homozygous mutations in *TYR* (c.934C>A, p.L312I) and *OCA2* (c.2363C>T, p.S788L) are strong candidates for disease-causing mutations in OCA in NHPs.

### 2.3. The TYR-L312I Mutation Results in Defective Melanin Synthesis

**Figure 5:**
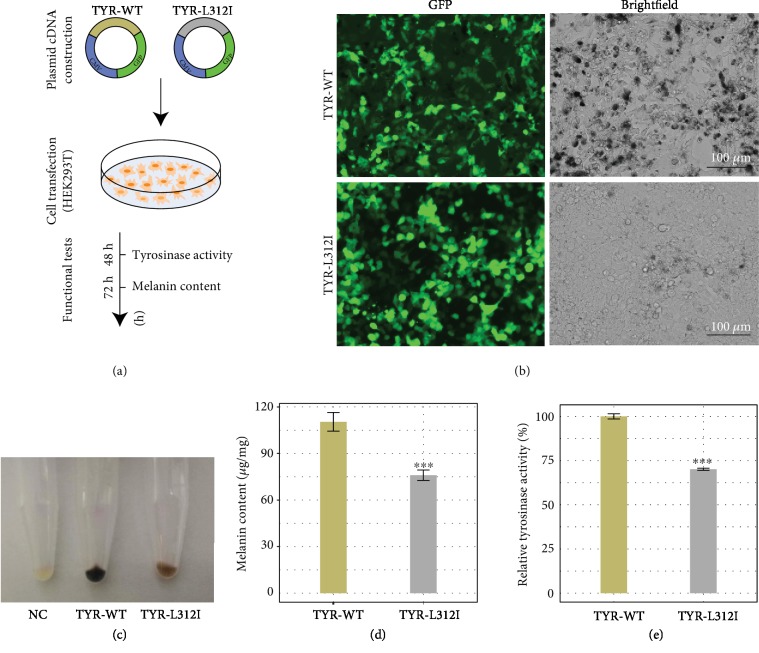
Functional assay of the TYR mutation. (a) Schematic diagram of the assay. (b) The transfection of mutated TYR into human HEK293T cells resulted in defective melanin synthesis. Scale bar, 100 *μ*m. (c) Cell pellets. NC: negative control. (d) Melanin content in HEK293T cells. (e) Tyrosinase activity. *N* = 3. ^∗^*p* < 0.05, ^∗∗^*p* < 0.01, and ^∗∗∗^*p* < 0.001.

To ultimately determine the pathogenicity of the identified mutation in *TYR*, we performed biological validation *in vitro*. Human HEK293T cells were transiently transfected with either the wild-type or mutant TYR gene (Figures 5(a) and 5(b)). After culture for 72 h, considerable melanin formation was apparent in 293T cells transfected with wild-type TYR, whereas a significant reduction in melanin was observed in the mutant cells (Figures 5(b) and 5(c)). Next, we examined tyrosinase activity and melanin content. We found that the TYR-L312I-transfected cells presented significant decreases in tyrosinase activity and melanin content in contrast to the same cells with wild-type TYR (Figures 5(d) and 5(e)). Taken together, we concluded that the TYR mutation identified in rhesus macaques with OCA led to impaired tyrosinase activity.

### 2.4. The OCA2-S788L Mutation Led to Reduced Production of Melanin

**Figure 6:**
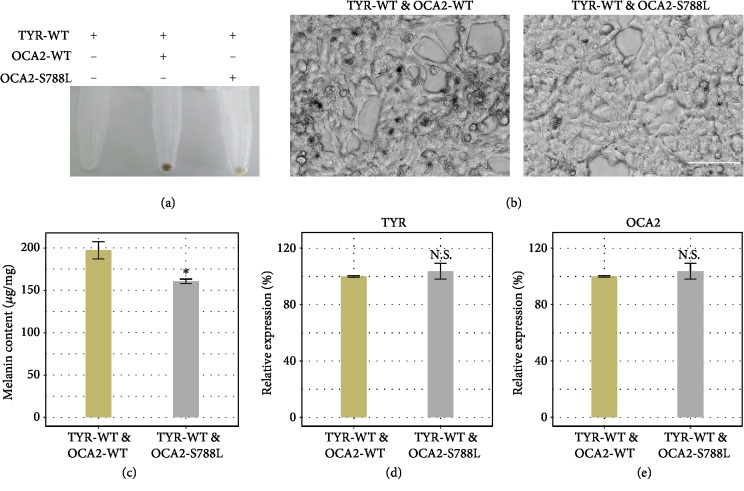
Functional assay of the OCA2 mutation. (a) Evaluation of cell pellets from the human HEK293T cell line transfected with TYR and OCA2. (b) Defect of melanin synthesis. (c) Measurement of melanin content. (d) Expression levels of the TYR and OCA2 genes in transfected HEK293T cells. *N* = 3. ^∗^*p* < 0.05, ^∗∗^*p* < 0.01, and ^∗∗∗^*p* < 0.001.

To elucidate the biological consequences of the OCA2 mutation identified in the albino macaques, we attempted to examine melanin formation following OCA2 knockdown by using shRNA and the cotransfection of OCA2 and TYR in HEK293T cells. In the OCA2-silenced HEK293T cells overexpressing wild-type TYR, no pigmentation was observed (Figure 6(a)). The simultaneous overexpression of wild-type OCA2 and wild-type TYR in the same cells resulted in apparent pigmentation, while the cotransfection of mutant OCA2 and wild-type TYR led to significant depigmentation (Figures 6(a) and 6(b)) and reduced melanin production (Figure 6(c)). We confirmed that there was no difference in expression levels when the cells were cotransfected with either TYR/OCA2 or TYR/mutant OCA2, excluding the deflected effects on melanin synthesis (Figures 6(d) and 6(e)). These results demonstrated that the OCA2 mutation (c.2363C>T, p.S788L) identified in the OCA monkeys could biologically affect melanin production.

## 3. Discussion

Because of their high genomic homology and similar anatomical eye structure and ontogenetic development of the fovea to humans, rhesus macaques present great potential as models for studying OCA and other neurological disorders [32, 33, 37–40]. In this study, we identified rhesus macaque models that were characterized by classical ocular characteristics comparable to those of OCA patients, including nystagmus, retinal hypopigmentation, and foveal hypoplasia. We also identified two homozygous missense mutations in the *TYR* (c.934C>A) and *OCA2* (c.2363C>T) genes and obtained biological evidence that each mutation significantly affects melanin synthesis via functional assays.

On the basis of the genetic predisposition concerning the *TYR* and *OCA2* genes, OCA has been classified into two types: OCA1 and OCA2 [6, 14]. OCA1 is further divided into subtypes OCA1A, characterized by the complete absence of melanin, and OCA1B, characterized by the accumulation of some pigments over time [8, 41]. The pathogenesis of OCA1A and OCA1B can be distinguished by the complete loss or partially reduced activity of tyrosine [8, 14, 41]. Partial pigmentation is also present in OCA2 patients [42]. The albino rhesus macaques identified in this study showed red hair and pink skin, indicating partial pigmentation. Through whole-genome sequencing, we identified two homozygous missense mutations in *TYR* (c.934C>A, p.L312I) and *OCA2* (c.2363C>T, p.S788L). Functional assays validated the impairment of melanin synthesis caused by each mutation. This evidence suggests that albino rhesus macaques can be genetically characterized as the OCA1B or OCA2 type. Two decades ago, Ding et al. discovered a rhesus macaque model of OCA1A with a possible mutation (p.S184X) in the *TYR* gene [43], and Prado-Martinez et al. reported an albino western lowland gorilla with a homozygous missense mutation (p.G518R) in *SLC45A2* [44]; however, the ocular phenotype was not described. In this study, we initially described the ophthalmologic clinical characteristics of the albino monkeys. As an experimental condition, the unevaluated retinal function of the albino monkeys will be assessed using electroretinography in future studies. The albino macaques identified in this study not only extend the known mutation spectrum of OCA in rhesus macaques but also provide new types of nonhuman primate models of OCA disease.

To date, hundreds of mutations in *TYR* and *OCA2* have been reported in OCA patients [24]; most of the relevant studies have determined mutation pathogenicity on the basis of cosegregation analysis or computational prediction. Few studies have included biological assays to validate the effect of the mutations on melanin synthesis. In pigmented cells, melanin is synthesized and sorted within a membrane-bound intracellular organelle, the melanosome, and the melanosomal maturation process includes four stages regulated by key melanogenic factors [28, 45, 46]. Recently, Song et al. transfected a plasmid carrying TYR cDNA into human HEK293T cells and observed melanin levels *in vitro* [47], indicating that the forced expression of TYR promoted melanin synthesis in HEK293 cells. Using this approach, we demonstrated that TYR mutation resulted in defective melanin synthesis. In addition, we developed a TYR/OCA2 coexpression approach to investigate the biological effect of OCA2 mutation on melanin synthesis in HEK293T cells, providing a simple assay for elucidating the functional impact of OCA2 mutation.

Great efforts have been made to develop new treatments for OCA disease [47–49]. Onojafe et al. treated a *Tyr^c-h/-ch^* mouse model with oral nitisinone, an FDA-approved inhibitor of tyrosine degradation, and proved that it could improve pigmentation in the fur, irises, choroid, and retinal pigment epithelia (RPE) [49–52]. Because mice lack a macula and fovea, they exhibit much lower spatial visual acuity than primates with a structural fovea [37]. Therefore, it remains a challenge to precisely evaluate the efficacy of nitisinone. The albinotic rhesus macaques identified in this study provide a model without this shortcoming and could be a useful candidate animal model. In human patients, Lee et al. evaluated the foveal changes in 44 children with OCA aged 0 to 6 years and provided evidence that photoreceptor layers continue to elongate at a reduced rate in albinism [53], indicating residual plasticity in the developing retina of patients with OCA and suggesting that earlier-stage treatment might result in a better outcome for these patients. This hypothesis could be tested in albino rhesus macaques. We have implemented an available breeding plan for these albino monkeys with the aim of generating a stable cohort of OCA NHPs for therapeutic development.

In the primate retina, melanin synthesis in the retinal pigment epithelium plays an important role in regulating the proliferation and differentiation of the neural retina [54]. Normal development of the foveal pit requires the absence of retinal vasculature inhabited by macula pigment [55]. In OCA patients, the higher the degree of foveal hypoplasia, the poorer the vision tends to be [56, 57]. In a recent study, Adams et al. reported that the number of letters read by five adult OCA1B patients showed a mild improvement after oral nitisinone treatment for 1 year [58]. It was hypothesized that if the physiological levels of melanin in the posterior segment of the eye could be maintained at early onset, foveal pit formation and visual acuity could be rescued in early-stage patients. To test this hypothesis, a preclinical trial in albino rhesus macaques will be required in the future [59–62]. The acceptable capacity of the cDNA lengths of the *TYR* and *OCA2* genes makes it feasible to conduct AAV-mediated gene therapy [63] and promote macular pigment formation in early-stage albinotic subjects. Another possibility is that the mutations could be precisely corrected *in vivo* through base-editing technology [45, 64]. For the cell transplantation strategy, RPE cells or RPE scaffolds with polarity are used as the cell source for replacing damaged RPE cells [63, 65–67]. These potential treatments could be tested preclinically in our NHP models with OCA.

In conclusion, we successfully identified novel rhesus macaque models of spontaneous oculocutaneous albinism with specific genetic mutations leading to defective melanin synthesis. This is the first well-established nonhuman primate model of oculocutaneous albinism, offering new opportunities for studies regarding disease mechanisms and therapeutic development.

## 4. Methods

### 4.1. Rhesus Macaques with Albinism

Rhesus macaques were maintained at the Institute of Medical Biology of the Chinese Academy of Sciences. This study was reviewed and approved by the institutional animal ethics committee. In total, we identified six rhesus monkeys with albinism, and clinical ophthalmic examinations were performed to evaluate their ocular phenotypes.

### 4.2. General Ophthalmic Examinations

Nystagmus is a common manifestation of OCA in human patients. To identify whether nystagmus existed in these albino rhesus macaques, we examined eye movement in the natural state. The anterior segment of the eyes was evaluated via slit lamp examination. Color fundus photography was performed using a CF-1 retinal camera (Canon, Japan). The NHP subjects were anesthetized with ketamine (10 mg/kg) and sodium pentobarbital (5 mg/kg). Their pupils were dilated with 0.5% tropicamide and 0.5% phenylephrine hydrochloride for 5 minutes. Eye drops consisting of artificial tears were applied to the corneal surface to avoid overdrying.

### 4.3. Spectral Domain Optical Coherence Tomography (SD-OCT)

High-resolution SD-OCT (Heidelberg Spectralis, Germany) was used to scan the retinal longitudinal section noninvasively. A horizontal b-scan across the fovea was also captured. For each b-scan, 25 scans were performed. Retinal thickness in the central or perimacular region was compared between albinotic subjects and healthy individuals.

### 4.4. Whole-Genome Sequencing and Genetic Analysis

Peripheral blood samples were obtained, and genomic DNA was extracted for the whole-genome sequencing (WGS) of the six albino NHPs and control NHPs. Briefly, 1 *μ*g of genomic DNA was sheared using ultrasound and then purified with AMPure XP beads. The shortened fragments underwent end repair and A-tailing, followed by the addition of index adaptors. After purification, the products were subjected to preamplification. Then, a library was constructed for high-throughput sequencing (HiSeq2500, Illumina). The sequencing results were aligned to the reference genome (rhesus macaque Mmul_8.0.1) by using the BWA program. The calling of single-nucleotide variants (SNVs) and indels was performed using ANNOVAR. Genetic variants were validated by Sanger sequencing (primers in Supplementary [Supplementary-material supplementary-material-1]).

### 4.5. Computational Assessment of Mutation Pathogenicity

To predict the pathogenicity of the identified mutations, a series of computational analyses were performed. First, multiple species alignments were performed using ESPript3.0. The three-dimensional homology modeling of the candidate proteins was analyzed by using SwissProt and visualized with the PyMOL algorithm. Then, the pathogenicity of the identified causative mutations was predicted using multiple bioinformatics programs, including MutationTaster, MutationAssessor, and SIFT, as described previously [68, 69].

### 4.6. Melanin Formation Assay *In Vitro*

We then asked whether the identified mutations impact melanin formation in human cells. Total RNA was extracted from the retinal pigment epithelium/choroid tissue using an RNeasy Mini Kit (Qiagen, 74204). Two micrograms of RNA was reverse transcribed into cDNA with M-MLV reverse transcriptase and random primers. The coding region of the *TYR* gene was amplified (primer sequences are provided in Supplementary [Supplementary-material supplementary-material-1]) and then cloned into the pLVX-IRES-ZsGreen1 vector. The coding sequence of *OCA2* was directly synthesized and then cloned into the pcDNA3.1 vector. Site-directed mutagenesis was performed by overlapping PCR (primer information in Supplementary [Supplementary-material supplementary-material-1]).

The HEK293T human kidney epithelial cell line was cultured in DMEM supplemented with 10% fetal bovine serum (Gibco), 100 U/ml penicillin, and 100 *μ*g/ml streptomycin at 37°C in an incubator with 5% CO_2_. The cells were seeded into 6-well plates and transfected using Lipofectamine 2000 according to the manufacturer's instructions. The pLVX-TYRwt-IRES-ZsGreen1 and pLVX-TYRmut-IRES-ZsGreen1 plasmids were transiently transfected into HEK293T cells. Human OCA2-targeted shRNAs (GCACTGTTGGGATGTGTTATCTTCAAGAGAGATAACACATCCCAACAGTGCTTTTTT) were cloned into pLent-U6-GFP-puro followed by transfection into 293T cells (Lipofectamine 2000). OCA2-arrested HEK293T cells were selected using puromycin (500 ng/ml). Transfected HEK293T cells were tested for melanin formation after 3 days of incubation as previously described [16, 17]. In brief, transfected cells were washed twice with phosphate-buffered saline (PBS, pH 7.4) and subsequently harvested using 0.05% trypsin and 0.02 EDTA. Thereafter, the cell pellets were lysed with 1 ml of 1 N NaOH containing 10% DMSO for 1 h at 80°C. After cooling, the lysates were centrifuged at 3000 rpm for 10 min, and the absorbance of the solutions at 405 nm was measured. The melanin content was quantified on the basis of a standard curve generated with synthetic melanin (Sigma).

### 4.7. Tyrosinase Activity Assay

The oxidation of L-DOPA to dopachrome was used to measure tyrosinase activity as previously described [16, 70]. In brief, cells were washed twice with PBS and then lysed with 50 mM PBS (pH 7.5) containing 1% Triton X-100 and 0.1 mM phenylmethylsulfonyl fluoride (PMSF). The reaction mixture containing 100 *μ*l of L-DOPA solution (5.0 mM in 50 mM PBS) and 100 *μ*l of cell extract was incubated at 37°C. The absorbance at 490 nm was measured using a microplate reader after incubation for 5 minutes, and tyrosinase activity was calculated from the obtained OD values.

### 4.8. Statistical Analysis

All results are presented as the mean ± SEM, and the statistical analysis was performed using Student's *t*-test and two-way ANOVA. A *p* value of less than 0.05 was deemed significant.

## Figures and Tables

**Table 1:** Basic clinical information of the albino rhesus macaques.

Monkey ID	Sex	Age (years)	Skin/hair color	Nystagmus
Ab-1	F	2	Pink/red	+
Ab-2	M	1	Pink/red	NA
Ab-3	M	1	Pink/red	+
Ab-4	F	3	Pink/red	+
Ab-5	M	6	Pink/red	+
Ab-6	M	10	Pink/red	+

Ab: albinism; F: female; M: male; +: positive; NA: not available.

## Supplementary Materials

Supplementary 1 Supplementary Video 1: nystagmus of the NHP model with oculocutaneous albinism.

Supplementary 2 Supplementary Video 2: nystagmus of another NHP model.

Supplementary 3 Supplementary Table 1: primers for identification of candidate mutations and overlay PCR. Supplementary Table 2: computational assessment of the missense mutations. The scoring results were based on each computational algorithm.
