# Supplementary material for: Nonhuman Primate Model of Oculocutaneous Albinism with TYR and OCA2 Mutations
Source: Research (Wash D C). 2020 Mar 11;2020:1658678. doi: 10.34133/2020/1658678 (PMC7086374; doi:10.34133/2020/1658678)
Supplement: Supplementary 3 — Supplementary Table 1: primers for identification of candidate mutations and overlay PCR. Supplementary Table 2: computational assessment of the missense mutations. The scoring results were based on each computational algorithm. [file 1658678.f3.pdf]

Supplementary Table S1 Primers for identification of candidate mutations and overlay PCR.

Items	Primer F (5´-3´)	Primer R (5´-3´)
TYR	CTACTGACTCAGGTGGTGAC	GATGGGTGCAGCACACCAAC
**OCA2**	ACAGAAGCTGACCACCAGG	CTCTCCTACACCACAATCTC
**mTYR-wt**	GAATTCATGATCCTGGCTGTTTTGTACTG	TCTAGATTATAAATGGCTCTGATACAACAAGTTG
**mTYR-mut**	GAACCCCAAGGATCCCCTCTTCAG	CTGAAGAGGGGATCCTTGGGGTTC
**mOCA2-wt**	GCTAGCATGCATCTGGAGGGCAGAG	TCTAGATTAATTCCATCCCACCACCAC
**mOCA2-mut**	CTGATTGGCGCGTTGGCAAACGTCGTGT	ACACGACGTTTGCCAACGCGCCAATCAG

Supplementary Table S2

Gene	AAChange	MutationTaster	MutationTaster_pred	RadialSVM	RadialSVM_pred
OCA2	c.C2363T:p.S788L	1	D	0.304	D
TYR	c.C934A:p.L312I	1	D	0.615	D

Gene	LR_score	LR_pred	MutationAssessor	MutationAssessor_pred	SIFT
OCA2	0.629	D	2.67	M	0.07
TYR	0.857	D	2.385	M	0.03

Gene	SIFT_pred	Polyphen2_HDIV	Polyphen2_HDIV_pred	LRT_score	LRT_pred
OCA2	T	0.994	D	0	D
TYR	D	0.008	B	0	N

Gene	FATHMM_score	FATHMM_pred	VEST3_score	CADD_raw	CADD_phred
OCA2	-1.06	T	0.948	3.981	20.4
TYR	-4.36	D	0.52	2.843	15.47
